# Drug-Induced Bullous Sweet Syndrome with Multiple Autoimmune Features

**DOI:** 10.4061/2010/176749

**Published:** 2010-08-24

**Authors:** Jared J. Lund, Erik J. Stratman, Deepa Jose, Ling Xia, Deborah Wilson, Mohammed Moizuddin

**Affiliations:** ^1^Department of Dermatology, Marshfield Clinic, 1000 N. Oak Avenue, Marshfield, 54449, USA; ^2^Department of Internal Medicine, Marshfield Clinic, 1000 N. Oak Avenue, Marshfield, 54449, USA; ^3^Department of Pathology, Marshfield Clinic, 1000 N. Oak Avenue, Marshfield, 54449, USA; ^4^Department of Rheumatology, Marshfield Clinic, 1000 N. Oak Avenue, Marshfield, 54449, USA

## Abstract

Sweet syndrome (SS) (Acute Febrile Neutrophilic Dermatosis) has been reported in association with autoimmune phenomena including relapsing polychondritis, drug-induced lupus, and the development of antineutrophil cytoplasmic antibodies (ANCAs). However, a combination of these autoimmune features has not been reported. Herein, we report a case of drug-induced bullous SS with ocular and mucosal involvement, glomerulonephritis, and multiple autoimmune features including clinical polychondritis with antitype II collagen antibodies, ANCAs, antinuclear (HEp-2), and antihistone antibodies in a patient on hydralazine and carbamazepine.

## 1. Introduction

Sweet Syndrome (Acute Febrile Neutrophilic Dermatosis) is characterized by rapid onset of fever, leukocytosis, and painful erythematous edematous papules, plaques, and nodules. Etiologies include infections, particularly from upper respiratory or gastrointestinal sources; inflammatory and autoimmune diseases; pregnancy; malignancy; drug; and idiopathic. Rare clinical manifestations include bullous lesions, oral involvement, glomerulonephritis, myositis, and ocular manifestations including conjunctivitis, episcleritis, and iridocyclitis [1–3]. Sweet syndrome (SS) has been associated with autoimmune phenomena including relapsing polychondritis, drug-induced lupus, and development of antineutrophil cytoplasmic antibodies (ANCAs). However, a combination of all these features has not been reported. Herein, we report such a patient.

## 2. Case Report

An 86-year-old female with bipolar disorder was admitted with anxiety, insomnia, fatigue, and acute renal failure. Although lithium levels were normal, lithium had been discontinued and replaced with carbamazepine 100 mg daily 2 days prior to admission. She was also taking hydralazine 100 mg three times daily for hypertension for 2 years with no dosage change in 8 months. On hospital day 8, she developed fever and conjunctivitis followed by oral erosions and painful lesions on her nose, ears, back, and fingers.

**Figure 1:**
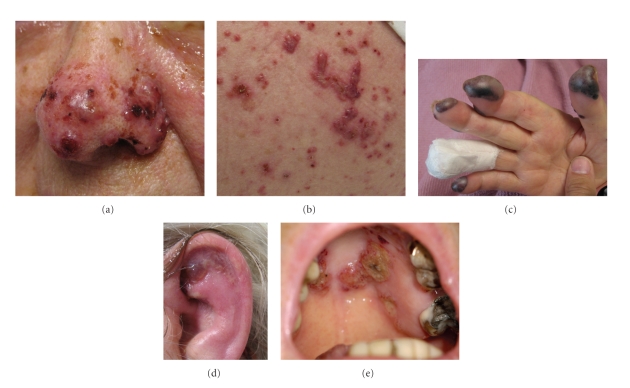
(a) Drug-induced Sweet Syndrome demonstrating tense vesicles and bullae with surrounding erythema over nose. (b) Tense inflammatory vesicles and bullae over central back. (c) Hemorrhagic bullae of distal finger pads and lateral fingers. (d) Erythema, edema, and focal bullous change overlying the cartilaginous portions of both ears. The non-cartilagenous lobes demonstrated no inflammatory changes. (e) Bullous lesions and erosions on the hard palate.

**Figure 2:**
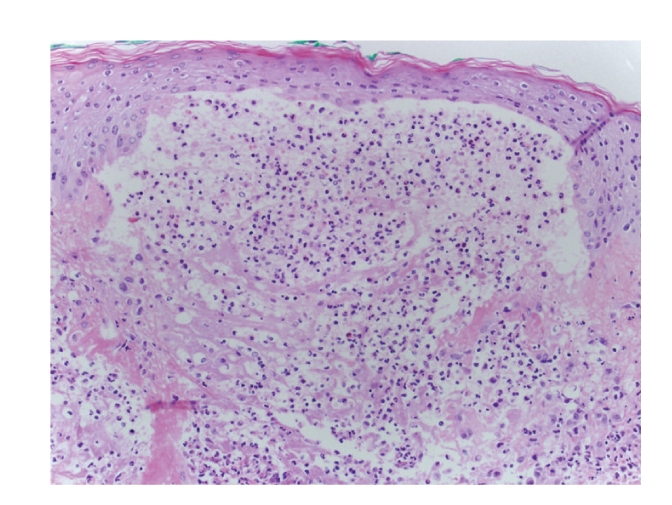
3 mm punch biopsy-upper back skin with focal subepidermal vesicles with neutrophilic microabscesses, perivascular and interstitial neutrophilic dermal infiltrate with leukocytoclasis 10x.

On examination, she appeared acutely ill and was febrile (38.4°C). Bilateral conjunctivitis with exudative discharge and periorbital edema was noted. Tense vesicles and bullae with surrounding erythema were noted on her scalp, nose, and back. The skin overlying the cartilaginous portions of both ears was erythematous and edematous with focal bullous change. The noncartilaginous lobes appeared normal. Erosions were noted on the hard palate and gingival mucosa (Figure 1). Tender hemorrhagic bullae were prominent on distal and lateral fingers (Figure 2). 

Laboratory testing revealed elevated C-reactive protein at 14 mg/dl (normal = 0 to 1 mg/dL) and erythrocyte sedimentation rate of 72 mm/hour (normal = 0 to 17 mm/hour). Her white blood cell count was normal at 5.5/mm^3^ (normal = 4.1 to 10.9/mm^3^) and hemoglobin was low at 9.7 gm/dL (normal = 11.7 to 15.5 gm/dL). Serum creatinine was 2.1 mg/dl (normal = 11.7 to 15.5 gm/dL). Serum creatinine was 2.1 mg/d and urinalysis demonstrated a new proteinuria (30 mg/dl) with hematuria (51 to 100 red blood cells/hp). Further labs showed positive AntiNuclear Antibody (HEp-2) with homogenous pattern of 1 : 640 (normal < 1 : 160). Anti-histone antibodies were elevated at 3.7 units (positive >1.5 units, Mayo Medical Laboratories). Perinuclear antineutrophil cytoplasmic antibodies (pANCAs) were positive to myeloperoxidase and proteinase 3 at 200 units/ml (normal = 0 to 9 units/ml) and 48.5 units/ml (normal = 0 to 3.5 units/ml), respectively. Anti-double-stranded DNA, anti-Smith, anti-RNP, SSA, SSB, SCL-70, or JO-1 antibodies were not detected, and complement levels were normal. Blood and urine cultures were negative. Serum protein electrophoresis showed acute phase reaction pattern.

Three-millimeter punch biopsies from the back and finger demonstrated focal subepidermal vesicles with neutrophilic microabscesses, perivascular and interstitial neutrophilic dermal infiltrate, and leukocytoclasis without vasculitis (Figure 2). Perilesional direct immunoflourescence (DIF) was negative. The patient declined ear cartilage biopsy, but anti-type II collagen antibodies were positive (47.6 EU/ml; normal <20 EU/ml; Mayo Medical Laboratories). 

**Figure 3:**
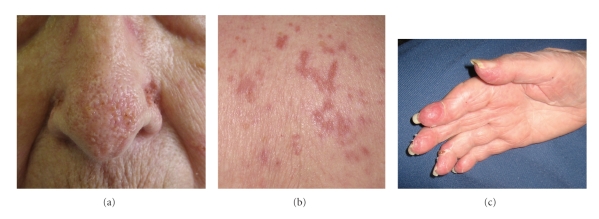
Clearing of affected areas 3 weeks following discharge on tapering dose of prednisone.

Drug-induced SS was suspected. Both carbamazepine and hydralazine were discontinued, and the patient was started on oral prednisone 60 mg daily. The patient declined renal biopsy. Her renal function, skin lesions, and mucosal lesions improved on prednisone, and she was discharged on a tapering dose. Skin lesions were resolved three weeks following discharge (Figure 3) Her MPO and PR-3 titers (57.7 units/ml and 11.1 units/ml, respectively) also decreased as her symptoms improved. The patient died of unknown causes 2 months following discharge, still on a tapering prednisone dose.

## 3. Discussion

The criteria for drug-induced Sweet Syndrome (SS) include the following [4]: an abrupt onset of painful erythematous plaques or nodules; histopathologic evidence of a dense neutrophilic infiltrate without evidence of leukocytoclastic vasculitis; pyrexia >38°C; temporal relationship between drug ingestion and clinical presentation, or recurrence after drug rechallenge; temporal relationship of resolution of lesions after drug withdrawal or treatment with systemic corticosteroids. In a review of 49 cases of drug-induced SS, leukocytosis was only documented in 11 cases [5]. Therefore, a normal white blood cell count should not rule out this diagnosis. Drug-induced SS occurs more often in women. The average time to onset of clinical symptoms after beginning drug therapy is 7.5 days [4, 5]. 

The pathophysiology of drug-induced SS is not clearly understood, and additional associated autoimmune features have been reported. Carbamazepine is known to cause drug-induced SS and drug-induced systemic lupus erythematosus (SLE) [6–8]. While carbamazepine is the most likely trigger, as onset of this therapy occurred 10 days prior to the development of our patient's cutaneous disease and fever, hydralazine was also considered, since hydralazine-induced SLE may occur 6 months to 6 years after starting the drug [9–12]. Since both drugs reportedly induce drug-induced SLE, a bullous variant of this disease must be considered in our patient. 

In reports of drug-induced bullous SLE, histopathologic examination demonstrated an interstitial neutrophilic infiltrate with subepidermal blister formation, interface change, and a positive DIF showing linear deposition of IgA and IgG at the basement membrane zone [13, 14]. Our patient had antihistone antibodies which may occur in drug-induced SLE [7], but the biopsy and clinical features best supports SS. Fever, oral erosions, and glomerulonephritis have all been reported in SS, but renal manifestations rarely occur in drug-induced SLE [8]. In this case, it is difficult to conclude that the patient's glomerulonephritis was directly related, as she was admitted with renal failure. Medications may induce SS with autoantibody profiles that mimic drug-induced SLE, but the skin eruptions lack characteristic histologic features of cutaneous lupus [12].

Sweet Syndrome has also been associated with relapsing polychondritis [15–22], usually in association with an underlying hematologic malignancy, dyscrasia, or solid malignancy. Our patient's clinical presentation of auricular inflammation that spared the non-cartilaginous lobes with anti-type II collagen antibodies supports the diagnosis of drug-induced autoimmune polychondritis. 

Cytoplasmic ANCA (c-ANCA) can occur in patients with SS [23]. Multiple cases of drug-induced SS in association with positive p-ANCA antibodies have also been reported [24, 25]. The presence of p-ANCA with anti-MPO antibodies appears to occur more commonly in drug-induced cases of SS [26]. ANCA may contribute to the pathogenesis of neutrophilic dermatoses [25].

## 4. Conclusion

Drug-induced SS is a unique dermatosis which may have unusual clinical and laboratory associations. Either carbamazepine or hydralazine may have incited this severe reaction. This case demonstrates unusual clinical features and multiple associated drug-induced autoimmune phenomena.

## Figures and Tables

**Table 1:** Drugs associated with Sweet's Syndrome.

Abacavir
All-trans retinoic acid
Bortezomib
Carbamazapine
Celecoxib
Clozapine
Diclofenac
Diazepam
Furosemide
Granulocyte colony-stimulating factor
Hydralazine
Imatinib
Lenalidomide
Minocycline
Nitrofurantoin
Norfloxacin
Ofloxacin
Oral Contraceptives
Pegfilgastrin
Propylthiouracil
Quinupristin/Dalfopristin
Trimethoprim-sulfamethoxazole
